# Genenames.org: the HGNC and VGNC resources in 2017

**DOI:** 10.1093/nar/gkw1033

**Published:** 2016-10-30

**Authors:** Bethan Yates, Bryony Braschi, Kristian A. Gray, Ruth L. Seal, Susan Tweedie, Elspeth A. Bruford

**Affiliations:** HUGO Gene Nomenclature Committee, European Molecular Biology Laboratory, European Bioinformatics Institute, Wellcome Genome Campus, Hinxton, Cambridge, CB10 1SD, UK

## Abstract

The HUGO Gene Nomenclature Committee (HGNC) based at the European Bioinformatics Institute (EMBL-EBI) assigns unique symbols and names to human genes. Currently the HGNC database contains almost 40 000 approved gene symbols, over 19 000 of which represent protein-coding genes. In addition to naming genomic loci we manually curate genes into family sets based on shared characteristics such as homology, function or phenotype. We have recently updated our gene family resources and introduced new improved visualizations which can be seen alongside our gene symbol reports on our primary website http://www.genenames.org. In 2016 we expanded our remit and formed the Vertebrate Gene Nomenclature Committee (VGNC) which is responsible for assigning names to vertebrate species lacking a dedicated nomenclature group. Using the chimpanzee genome as a pilot project we have approved symbols and names for over 14 500 protein-coding genes in chimpanzee, and have developed a new website http://vertebrate.genenames.org to distribute these data. Here, we review our online data and resources, focusing particularly on the improvements and new developments made during the last two years.

## INTRODUCTION

The HUGO Gene Nomenclature Committee (HGNC) is the only internationally recognized authority for assigning standardized nomenclature to human genes. It is crucial that there are user-friendly and unambiguous ways of referring to human genes that can be used by scientists in publications, presentations and when searching biomedical databases, and equally by clinicians and their patients; in short everyone needs a common language for human genes. The HGNC fulfils this requirement by providing a unique gene symbol and corresponding name for protein-coding and non-coding RNA genes and pseudogenes in the human genome. HGNC's standardized gene symbols are a core component of human genomics, enabling integration of information and facilitating discussion, collaboration and discovery.

All of the HGNC's public data, tools and accompanying help documentation can be accessed via the http://www.genenames.org website. This site provides an easy access portal to an abundance of information about a human gene, including links to genome browsers, nucleotide and protein sequence data, orthologs in other species, gene family membership, relevant publications, variation data and clinical associations.

For other vertebrate species that have their own nomenclature groups the HGNC work closely with them to ensure that orthologous genes are assigned equivalent symbols whenever possible. However, as nomenclature committees currently exist for only six other vertebrate species we have established a sister project, the Vertebrate Gene Nomenclature Committee (VGNC), which assigns names to vertebrate species lacking a dedicated gene nomenclature group. Following our pilot project using the chimpanzee genome, standardized nomenclature has been assigned to over 14 500 chimpanzee genes. These data are available from our new website http://vertebrate.genenames.org, and over 10 000 of these VGNC gene symbols and names can be seen associated to chimpanzee genes in the NCBI Gene (1) and Ensembl (2) databases.

## DATA

As of September 2016, we have 39 870 active entries within our HGNC database (3) of which 19 017 are for protein-coding genes. Though the total number of protein-coding genes has been relatively stable over the last three years, there has been significant fluctuation in locus type within our gene set, with over 200 entries being reclassified to or from the protein-coding locus type as new evidence has become available. As well as locus type classification changes our gene entries are also regularly being reviewed and updated with additional cross references, and nomenclature revisions. The majority of HGNC's human gene symbols have become or are becoming well entrenched in the literature and databases, and should never require reassignment. HGNC limit the number of symbol alterations and only ever change symbols for specific reasons, the most common being if a symbol can be updated from an uninformative placeholder designation, such as our C$orf#s, KIAA# or FAM#s, or if the symbol was originally assigned based on information that has since been found to be erroneous and the existing symbol could be misleading. To avoid symbol changes but improve our nomenclature we can make updates to gene names while retaining the symbols, for example, to remove references to other species or human-specific phenotypes.

Prior to gene nomenclature assignment and updates HGNC curators review the recent literature and so are in a good position to fill obvious gaps in the gene ontology (GO) (4) terms associated with a gene. As one of our curators has extensive experience in GO annotation we now contribute to the human GO annotation effort in a limited way; particularly for genes that previously lacked GO annotation, we aim to ensure that any experimental evidence used to support a new name is reflected in the functional annotation for the gene. HGNC-assigned GO annotations are available from the EBI QuickGO browser (5) and the GO consortium AmiGO 2 browser (6). The HGNC also continue to be active members of the consensus coding sequence (CCDS) consortium (7), collaborating with NCBI's RefSeq (8), Wellcome Trust Sanger Institute's HAVANA (9) annotation group, Ensembl and the Mouse Genome Database (10). As well as giving HGNC input into the decision making process for the human CCDS gene set, this also ensures that our named protein-coding genes accurately reflect the current expert manual annotations provided by these groups.

The vast majority of new symbols being assigned in recent years belong to long non-coding RNA genes and pseudogenes. Long non-coding RNAs are named based on either their genomic location or published (or pre-publication) functional data, as for protein-coding genes. The number of publications describing novel lncRNA genes has increased markedly in the last few years and this is reflected in the number of symbol requests we receive for lncRNA genes, which now comprise over half of all requests received. Naming annotated pseudogenes not only provides information on the origin of the locus that an anonymous database identifier cannot, but also provides landmarks in genomic space, for example, for describing genome-wide association study loci in protein-coding gene deserts.

**Figure 1.**
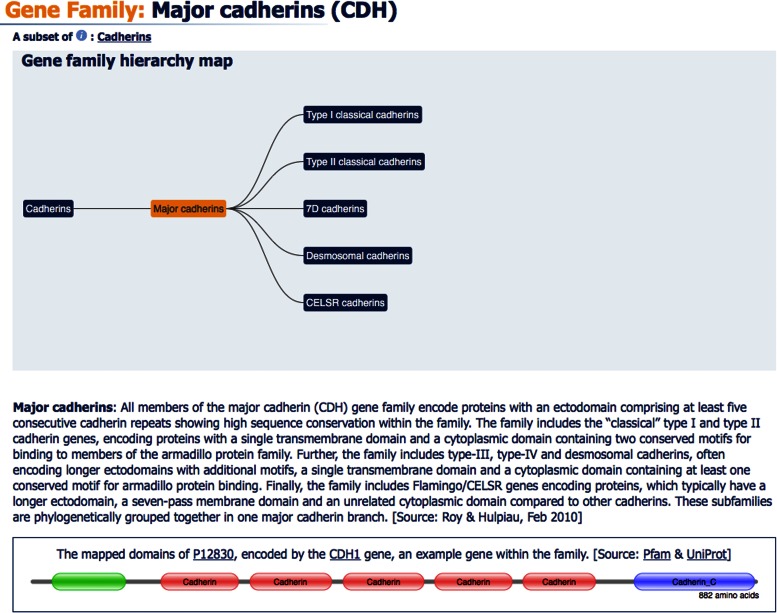
A section of the ‘Major cadherins’ gene family report page, showing a gene family hierarchy map, the gene description and the protein domain structure of a representative gene within this family.

Gene family data have been a major focus of our recent curation work, with the number of curated gene families increasing from 588 in 2014 to over 1000 in 2016. Genes are grouped into families based on homology or a shared characteristic such as a common function and/or phenotype, or membership of a complex. We have also begun curating the relationships between different gene families, for example, the protein phosphatase gene family members represent a subset of the phosphatases gene family. As the relationships between gene families may span multiple levels, and it is possible for an individual family to be a subset of more than one gene family, it is best to view these relationships as a hierarchy. Our database has been redesigned to better capture these complex gene family relationships and new web displays have been developed to allow their visualization (Figure 1). For a full review of our gene family resources please see our recent article (11).

Partly inspired by HGNC's gene families, FlyBase recently introduced manually curated ‘Gene Groups’ for Drosophila (12), and HGNC and FlyBase curators have collaborated to produce reciprocal links between these resources to enable users to find relevant genes in another species. This can be particularly helpful in cases where the nomenclature of the genes differs considerably between species. It should be noted that the mappings do not always represent sets of orthologous genes, for example, broadly equivalent complexes between species can have different specific subunit compositions. HGNC and FlyBase are still adding many new families/groups and the work to represent a complete mapping between resources is ongoing. The reciprocal links are provided on the HGNC gene family report pages and FlyBase gene group reports are updated with each FlyBase release.

Naming genes in other species in line with human genes has been an accepted concept for many years, and was first formalized at the International Society for Animal Genetics meeting of 1990 where members unanimously agreed that gene naming in animals should follow the rules for human gene nomenclature (13), including the use of identical symbols for homologous genes. Many vertebrate species now have a sequenced genome but no dedicated nomenclature committee, and so the annotated genes are named according to the rules of the database they are found in. This results in problems such as different names being assigned to the same gene, anonymous identifiers being assigned and misnaming of highly related genes. To avoid this confusion in gene naming in such species we have instigated the VGNC, which aims to ensure that genes across vertebrate species are named in a consistent and standardized manner in line with human gene nomenclature.

Considerable effort has been applied to standardising and simplifying our gene names prior to their transferral to other species, with over 4000 edits being made to human gene names (but not symbols) in 2015. This included the removal of species names from genes originally named based on an orthologous gene, and the removal of aliases, superfluous information and punctuation. In addition to changes made to gene names we have also made some limited changes to gene symbols, including reassigning human phenotype-based gene symbol designations that would make little or no-sense for non-human orthologs. For example, we renamed *CIRH1A* (cirrhosis, autosomal recessive 1A) to *UTP4* (UTP4 small subunit processome component) and *KAL1* (Kallman syndrome 1 sequence) to *ANOS1* (anosmin 1), based on their established use in the literature and community agreement.

## WEBSITE

Our primary website http://www.genenames.org provides a public access portal to all of our human specific data. Our data set is updated nightly, and is freely available via our download tools and BioMart (14) and RESTful web services. The last major redesign of this site was released in May 2011, and since then we have continued to build upon this, updating and creating new resources to search, browse and download our data. A brief outline of the changes and additions made to the site since our 2014 publication (15) follows.

### Gene family reports

In 2015, we restructured our gene family report pages to support the visualization and browsing of complex relationships between families (Figure 1). Each gene family has its own report page that follows a standard format and contains a list of genes in that family, along with the option of links to further data such as relevant publications, a gene family description, a hierarchy map showing the relationship to other families, the domain structure of an example family member and curators’ comments.

#### Gene family hierarchy maps

Gene families may be arranged in complex hierarchies that feature many different levels, with each level having its own family report. These levels can be viewed using our gene family hierarchy maps. A hierarchy map illustrates the path through the hierarchy, centred on a selected gene family and only shows those families that are directly related. The full hierarchy can be seen by selecting the family at the highest level within the map. As well as being a visual representation of the relationship between different gene families these maps also act as a navigation tool, allowing users to jump to the family report page of any of the gene families shown.

#### Representative protein domain map

If the gene products of a gene family share a typical domain structure or even a single domain, we select an ‘example’ gene to illustrate the domain(s) via a graphical display which is sourced from Pfam (16) via a UniProt (17) ID.

#### Gene family descriptions and comments

Gene family reports often feature a description of the family, which may be sourced from Wikipedia (https://en.wikipedia.org), UniProt, a publication, or may be written by an HGNC curator or specialist advisor. Curators may also include explanatory remarks about the family in a ‘Comments’ field. These comments typically relate to the basis for including or excluding genes when there are conflicting opinions in the literature; this is particularly pertinent to the composition of some protein complexes. This field is also used to highlight non-functional family members and to direct users to other relevant gene families that are not linked within the hierarchy map.

#### Downloading gene family data

Gene family reports that are not within a hierarchy, or that are at the lowest level of a hierarchy, display a table listing all genes that are family members. Gene family pages that represent higher levels within a hierarchy will only display a table of gene family members if genes at that particular level do not feature in any of the lower levels of the family hierarchy. Where a gene family is further divided into subsets, users can opt to view a table listing all of the genes that are contained within subsets of that family. The data in these tables may be downloaded as a tab separated text file from the bottom of each family report page.

#### Searching gene family data

We have added an extra ‘Gene family search’ option to the type of search that can be selected from our sitewide search facility. Users can now opt to search all aspects of the site, gene symbols only, gene families only or just static pages such as the help and news pages, using the drop-down menu next to the search box. If a search is conducted using the default option of ‘Search everything’ it is now possible to facet the search results by each of the available search options.

### Data downloads

Our existing BioMart server http://biomart.genenames.org has been updated to the latest version 0.9.0, and now hosts two ‘HGNC mart’ portals. The first of these is the ‘Gene’ portal for gene-centric queries that replaced our existing mart service, while the second is a new ‘Family’ portal designed specifically to enable querying and downloading of our curated gene family data. As with the previous version, BioMart provides a user-friendly graphical interface capable of building complex queries to create custom data sets that can then be downloaded as a tab separated text file. In addition to the graphical interface the BioMart server can also be queried programmatically using the SPARQL query language or REST/SOAP web services. Currently BioMart is our only download application that allows users to select and query our gene family data, but we plan to incorporate gene family data into our increasingly popular RESTful web service in the near future.

The changes to our gene family resources have resulted in further minor changes to the download options we provide. Our custom downloads tool and gene mart portal now include ‘Gene Family ID’ and ‘Gene Family Name’ as attributes that can be selected, and we provide a complete ‘HGNC Gene Family data set’ file in either tab separated text or JSON format from our statistics and downloads page.

### Reusable javascript components

Several of the improvements made to the HGNC website have resulted in the development of stand-alone JavaScript components that we believe may be useful to other projects. We have created a public library on GitHub https://github.com/HGNC from which these components may be downloaded and reused without restriction. Currently we have four such components: europe-pmcentralizer, used within our gene symbol and gene family reports to convert a PubMed identifier into a reference, based on data retrieved asynchronously from Europe PMC (18); pfam-dom-draw used on gene family report pages to take an HGNC approved symbol and its linked UniProt accession to create a stylized diagram of the protein, marking up the locations of any Pfam domains; and two components that relate to the drawing of the gene family hierarchy maps discussed previously. The first of these components, iHAG, draws a horizontal acyclic graph based on data passed to it via a JavaScript object. The second component, hgnc-gene-family-mapper builds on iHAG to draw a complete gene family hierarchy map when given a gene family ID as input.

### VGNC website

**Figure 2.**
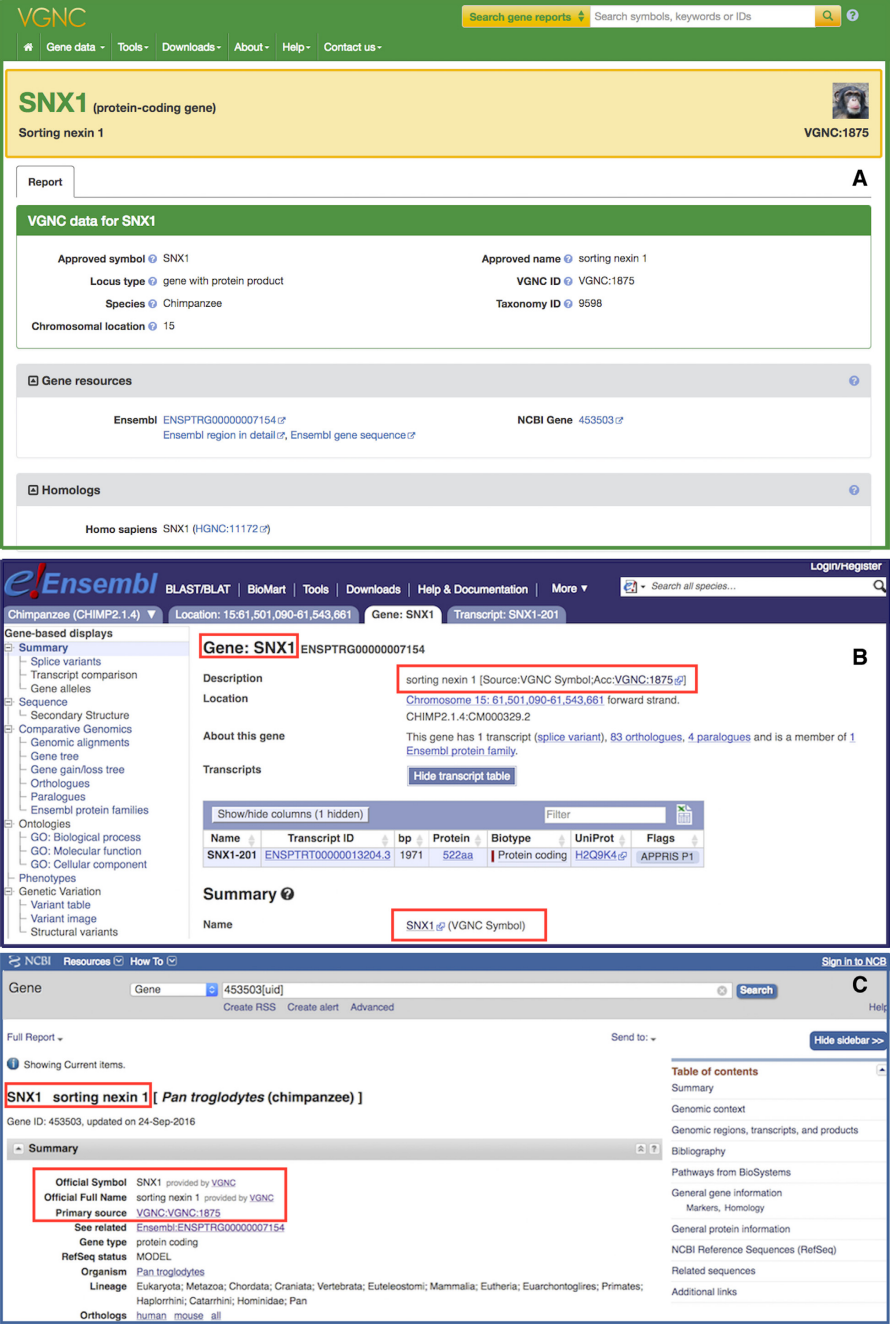
Section (**A**) shows a sample gene symbol report from the VGNC website. Sections (**B**) and (**C**) show the inclusion of VGNC gene symbols and names in the Ensembl and NCBI gene resources, respectively.

In July 2016 we launched our new VGNC site for vertebrate nomenclature data, accessible at http://vertebrate.genenames.org and also via a new VGNC menu in the header of the HGNC site. The VGNC site shares consistent branding with the HGNC site and replicates some of the displays provided for the HGNC data. Each webpage has a header containing links that enable browsing of the various sections of the website and a footer that provides a site map, allowing a user to jump quickly to a specific page of interest. VGNC symbols and names for more than 10 000 chimpanzee genes are now included in both the NCBI Gene and Ensembl resources (Figure 2) along with reciprocal links to the VGNC site.

#### Searching the VGNC site

The VGNC home page contains a large search box to allow quick navigation to the content of interest. As with the HGNC site the VGNC site utilizes Solr to power its search application. Hint text is provided within the search box to indicate some of the accepted search terms, which include approved gene symbols, keywords likely to be found within a gene name such as kinase or pseudogene, as well as gene identifiers from VGNC, NCBI Gene and Ensembl. Further information on the types of search query that are supported can be found on the search help page http://vertebrate.genenames.org/help/search. In addition to the large search box on the homepage a search box forms part of the header on all other pages, meaning that the search application is always easily accessible.

Search results are displayed in order of significance with the most relevant result being listed first. The first line of a result contains the VGNC approved gene symbol followed by the approved gene name. Below this we display the VGNC ID, the locus group the gene belongs to and the species it is from. Each result links to a symbol report for that particular gene.

#### VGNC gene symbol reports

As with http://www.genenames.org the main interface to the manually curated data is the gene symbol report pages. Each gene symbol report follows the same consistent, easy to read format. At the top of each report is a ‘quick reference’ box containing the key summary information for that gene, including approved gene symbol, approved gene name, gene locus type, VGNC identifier and an icon representing the species the gene belongs to. Below this is a box containing all the information that is curated by the VGNC in addition to the fields found in the quick reference section, including a chromosomal location, a taxonomy identifier and, where applicable, any previous or alias gene symbols and names. Context-sensitive help is provided for each of these fields by means of a small help icon next to the field label.

Further information on the gene is displayed in a series of collapsible panels. These panels group together external references from related resources; for example, all of the links for this gene in other gene-centric resources or genome browsers appear together in a ‘Gene resources’ panel. Organizing our external references in this manner allows a user to quickly identify links that are of particular interest to them. All manually curated external references are tagged with a ‘curated’ icon.

#### Downloading VGNC data

A key requirement of the VGNC project is easy dissemination of the curated nomenclature data to other genomics resources. Therefore, we provide a statistics and downloads page http://vertebrate.genenames.org/download/statistics-and-files that allows the download of files containing all of our curated gene symbol data in either JSON format or tab delimited text format. It is also possible to download subsets of the data broken down by any combination of species, chromosome, locus group and locus type.

### Future directions

We will continue the systematic naming of all newly identified human protein-coding genes, non-coding RNA genes and pseudogenes, including novel genes annotated on new alternative haplotypes of the human genome. As part of our ongoing review of the biomedical literature, we will reassign nomenclature for human genes that were initially assigned temporary placeholder symbols as more information allowing the characterization of these genes becomes available. In addition to this we will continue our efforts to simplify and standardize human gene nomenclature to ensure that it is suitable for use across vertebrate species where appropriate.

In 2017, we plan to release a new version of the HGNC website. This will represent significant changes to both the frontend and backend code running the website. The frontend code will be re-implemented using modern web technologies including AngularJS, HTML5, JQuery and the Bootstrap framework. Full functionality of the existing site will be retained, with the redesign aiming to re-skin rather than radically change the existing views. Revamping the site in this way will allow us to modernize without alienating our existing users, making the site quicker, more efficient and also more tablet- and mobile-friendly, reflecting the increasing use of mobile devices to access our data. The new HGNC site will first be trialled as a beta release to allow any changes resulting from user feedback to be made prior to replacing the existing site.

The new design principles outlined above are already in use on our VGNC website and much of the code developed will be reused for the new HGNC site. The next 12 months will see a rapid expansion of the VGNC website, with nomenclature data for several new species including cow and dog being added, and new data displays and tools being implemented to mirror the functionality provided by the HGNC website. VGNC data will also be added to the existing HGNC RESTful and BioMart services, providing a single unified way of accessing all the nomenclature data that we curate. Please email us at vgnc@genenames.org with suggestions of a species for gene naming or for help naming gene families or individual genes in any vertebrate.
